# Garcinol-Attenuated Gastric Ulcer (GU) Experimentally Induced in Rats Via Affecting Inflammation, Cell Proliferation, and DNA Polymerization

**DOI:** 10.7759/cureus.43317

**Published:** 2023-08-11

**Authors:** Yousef F Alatawi, Marwan A Alhablani, Fahad A Al-Rashidi, Waleed S Khubrani, Salman A Alqaisi, Hanan M Hassan, Mohammed M Al-Gayyar

**Affiliations:** 1 PharmD Program, University of Tabuk, Tabuk, SAU; 2 Pharmacology and Biochemistry, Delta University for Science and Technology, Gamasa, EGY; 3 Pharmaceutical Chemistry, Faculty of Pharmacy, University of Tabuk, Tabuk, SAU; 4 Biochemistry, Faculty of Pharmacy, Mansoura University, Mansoura, EGY

**Keywords:** tumor necrosis factor (tnf)-α, proliferating cell nuclear antigen (pcna), mammalian target of rapamycin (mtor), interleukin (il)-1β/4/10, cyclooxygenase-2 (cox2), cyclin d1

## Abstract

Background: Gastric ulcer (GU) is one of the most critical gastrointestinal tract disorders. Garcinol is a polyisoprenylated benzophenone in Garcinia fruit with antioxidant and anti-inflammatory priorities.

Objectives: We aimed to assess the protective effects of garcinol against GU induced in rats. We investigated garcinol’s effects on DNA polymerization via mammalian targets of rapamycin (mTOR) and cyclin D1, cell proliferation via proliferating cell nuclear antigen (PCNA), inflammatory pathway via cyclooxygenase-2 (COX2), TNF-α, and IL-1β, and anti-inflammatory pathway via IL-4 and IL10.

Methods: In our study, we administered a single oral dose of 80 mg/kg of indomethacin to rats to induce GU. Some of the rats were given a treatment of 50 mg/kg of garcinol. We examined the expressions of mTOR, cyclin D1, PCNA, COX2, TNF-α, and IL-1β/4/10 in the gastric tissues. Furthermore, we stained sections of the gastric tissues with Masson trichrome.

Results: The areas of gastric tissues in the GU group showed severe hemorrhage and extensive fibrosis. Treating GU rats with garcinol prevented bleeding and ameliorated the fibrosis caused in gastric cells by GU. Moreover, treatment with garcinol significantly decreased the expression of mTOR, cyclin D1, PCNA, COX2, TNF-α, and IL-1β associated with elevation of IL-4 and IL-10.

Conclusion: Garcinol has been found to provide therapeutic benefits in rats with induced GU. These benefits may be due to its ability to decrease the expression of DNA polymerization markers, cell proliferation markers, and inflammatory markers at the gene and protein levels.

## Introduction

Peptic ulcer disease, which includes both stomach and duodenal ulcers (DUs), has been a severe threat to the global population for the past 200 years, with a high morbidity and significant death rate. There are two main reasons for gastric ulcer (GU) incidence: *Helicobacter pylori* infection and consumption of non-steroidal anti-inflammatory drugs [1]. Epidemiological data show that there are notable differences in the incidence and frequency of GU and its consequences across different regions. DU is the primary form in the Western population, while GU is more common in oriental countries [2]. Previous reports have shown that the rate of death from proliferated peptic ulcers ranges between 1% and 24% [3]. However, GU is associated with a higher risk of mortality due to hemorrhage, perforation, and obstruction. GU treatment options include nonoperative and operative procedures. The former includes proton pump inhibitors, histamine-2 receptor antagonists, antacids, and cytoprotective agents, while the latter involves simple sutures and laparoscopy with better results from laparoscopy [4]. Definitive surgery remains an important option for managing gastric perforation, even without concrete evidence linking it to Helicobacter pylori. The success of the surgery depends on the patient's preoperative condition rather than the specific type of surgery [5]. However, therapeutic agents for treating GU may lead to side effects such as gastrointestinal disturbances, heart failure, hypertension, renal failure, cirrhosis, hepatic impairment, and osteoporosis [6]. Therefore, there is a need for new, safe therapeutic agents to treat GU.

Over the past few decades, researchers have focused their efforts on studying various plants with the hope of discovering therapeutic effects against chronic diseases. One such plant is Garcinia cambogia and Garcinia indica, small tropical trees in different regions of Asia, Africa, and the Pacific. These plants have been found to contain several active constituents, including hydroxycitric acid, hydroxycitric acid lactone, garcinol, Cambogia, isogarcinol, guttiferones, and xanthochymol [7]. Among these active constituents, garcinol, which has a structure resembling that of curcumin due to the β-diketone group, has been proven to possess anti-oxidative, anti-inflammatory, and anti-cancer properties [8]. However, only one previous study has shown garcinol's ability to attenuate GU via free radical scavenging activities [9]. Therefore, we conducted a survey to experimentally assess the protective effects of garcinol against GU in rats. We examined garcinol's impact on DNA polymerization markers (mTOR and cyclin D1), cell proliferation markers (PCNA), inflammatory markers (COX2, TNF-α, and IL-1β), and anti-inflammatory markers (IL-4 and IL-10).

This article's contents were presented as a meeting abstract at the 2023 Dubai International Pharmaceuticals and Technologies Conference and Exhibition (DUPHAT) held from January 10 to January 12, 2023. Additionally, it was also shared at the Third International Conference on Engineering and Applied Natural Sciences (ICEANS 2023) in Konya, Turkey from January 14 to January 17, 2023.

## Materials and methods

Animals and treatment outlines

The research study included 30 Sprague Dawley rats that weighed between 180 and 200 g. The Faculty of Pharmacy at Delta University for Science and Technology (FPDU) Research Ethics Committee approved the research protocol, and approval No. (FPDU19/2022) was granted. The rats were divided into three groups.

The Control Group (10 Rats)

The rats were deprived of food for 24 h but had access to water. They were administered 0.5% carboxymethylcellulose (CMC) via oral gavage and left untreated throughout the experiment.

The GU Group (10 Rats)

The rats were deprived of food for 24 hours but had access to water. Afterward, they were given 80 mg/kg of indomethacin orally to induce GU [10].

The GU Treated With Garcinol Group (10 Rats)

Following the induction of GU, the rats received a daily oral gavage of 50 mg/kg of garcinol (obtained from Santa Cruz Co in Dallas, TX) for a period of seven days.

Previous research has explored the effectiveness of garcinol in treating GU in rats, using a dosage of 200 mg/kg [9]. We conducted our own preliminary studies, testing garcinol at four different concentrations: 50, 100, 150, and 200 mg/kg. Following a thorough analysis, we determined that a dosage of 50 mg/kg was the most beneficial and yielded positive therapeutic results.

Collection of samples from rats

First, animals were sacrificed, then the whole stomach of each rat was removed and weighed. Followed by the fixation of a piece of each stomach in 10% buffered formalin and subsequently used to illustrate the morphologic and immunohistochemistry investigations. Another piece of each stomach was homogenized in a 10-fold sodium-potassium phosphate buffer, filtered, and the supernatant was stored at -80°C.

Morphologic analysis and immunohistochemistry

To assess the extent of fibrotic tissue, sections of the stomach measuring five micrometers were cut and stained with Masson trichrome stain. The fibrotic tissue scores were determined by examining at least 10 fields in each animal section stained with Masson Trichrome at high power. Additionally, we used monoclonal anti-proliferating cell nuclear antigen (PCNA) from Sigma Aldrich Chemicals Co., St. Louis, MO to immune-stain other sections. This was done at 4°C and as described previously by our group [11-13].

Enzyme-linked immunosorbent (ELISA) assay

To detect mammalian targets of rapamycin (mTOR), cyclin D1, PCNA, cyclooxygenase-2 (COX2), IL-4, IL-10, tumor necrosis factor (TNF)-α, and interleukin (IL)-1β, ELISA kits purchased from MyBioSource, Inc. (San Diego, CA) and USCN Life Science Inc. (Houston, TX) were used in accordance with the manufacturer's instructions.

Quantitative real-time polymerase chain reaction (RT-PCR)

Our group has previously conducted an analysis of rat gastric lysate to determine the levels of mRNA for genes such as mTOR, cyclin D1, PCNA, COX2, TNF-α, IL-1β, IL-4, and IL-10 as described previously [14-17]. To maintain consistency, glyceraldehyde-3-phosphate dehydrogenase (GAPDH) was utilized as a housekeeping gene and internal reference control. The PCR primers employed for each gene have been summarized in Table 1.

**Table 1:** Primer sets used to detect gene expression in rats.

Gene symbol	Primer sequence	Accession number
GAPDH	F: 5`-CCATCAACGACCCCTTCATT-3` R: 5`-CACGACATACTCAGCACCAGC-3`	NM_017008.4
mTOR	F: 5′-CTGCACTTGTTGTTGCCTCC-3′ R: 5′-ATCTCCCTGGCTGCTCCTTA-3′	NM_019906.2
Cyclin D1	F: 5′-TCGACGGCCATTACCAATCG-3′ R: 5′-CGCAGACCTCTAGCATCCAG-3′	X75207.1
PCNA	F: 5′-AGTTTTCTGCGAGTGGGGAG-3′ R: 5′-AAGACCTCAGAACACGCTGG-3′	NM_022381.3
COX2	F: 5′-TGACTGTACCCGGACTGGAT-3′ R: 5′-CATGGGAGTTGGGCAGTCAT-3′	NM_017232.4
TNF-α	F: 5`-AAATGGGCTCCCTCTCATCAGTTC-3` R: 5`-TCTGCTTGGTGGTTTGCTACGAC-3`	X66539
IL-1β	F: 5′-TTGAGTCTGCACAGTTCCCC-3′ R: 5′-GTCCTGGGGAAGGCATTAGG-3′	NM_031512.2
IL-4	F: 5`-GTACCGGGAACGGTATCCAC-3` R: 5`-GTGAGTTCAGACCGCTGACA-3`	X16058.2
IL-10	F: 5`-CATTCCATCCGGGGTGACAA-3` R: 5`-GTAGATGCCGGGTGGTTCAA-3`	L02926.1

Statistical analysis

When displaying quantitative variables, we utilize mean ± standard error. To identify significant differences between groups under study, we utilize a one-way analysis of variance (ANOVA), followed by a post hoc Bonferroni correction test. Excel is the software used to perform all statistical analyses. We consider a value of p < 0.05 to indicate statistical significance.

## Results

Protective effects of garcinol against GU-induced in rats

**Figure 1:**
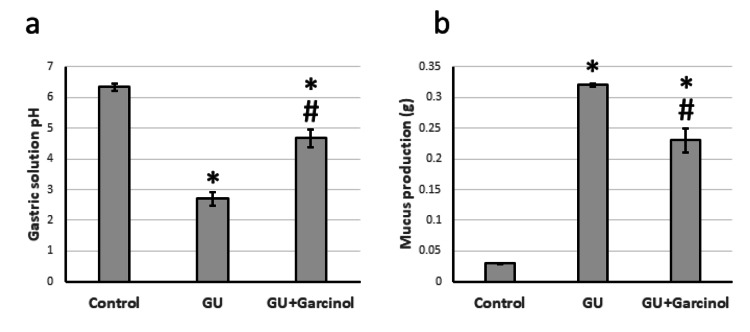
The effect of GU and administration of 50 mg/kg garcinol on the pH of the gastric solution (a) and the production of mucus in the stomach (b). ^*^Represented a significant difference for the group as compared with the control group at p<0.05. ^#^Represented a significant difference for the group as compared with the GU group at p<0.05. GU, gastric ulcer

After conducting our study, we observed that the GU group had a notable decrease in gastric solution pH and a significant rise in gastric mucous secretion compared to the control group. However, administering garcinol to GU rats effectively reversed these effects, as shown in Figure 1.

Effect of garcinol on GU-induced alteration in gastric tissues morphology

**Figure 2:**
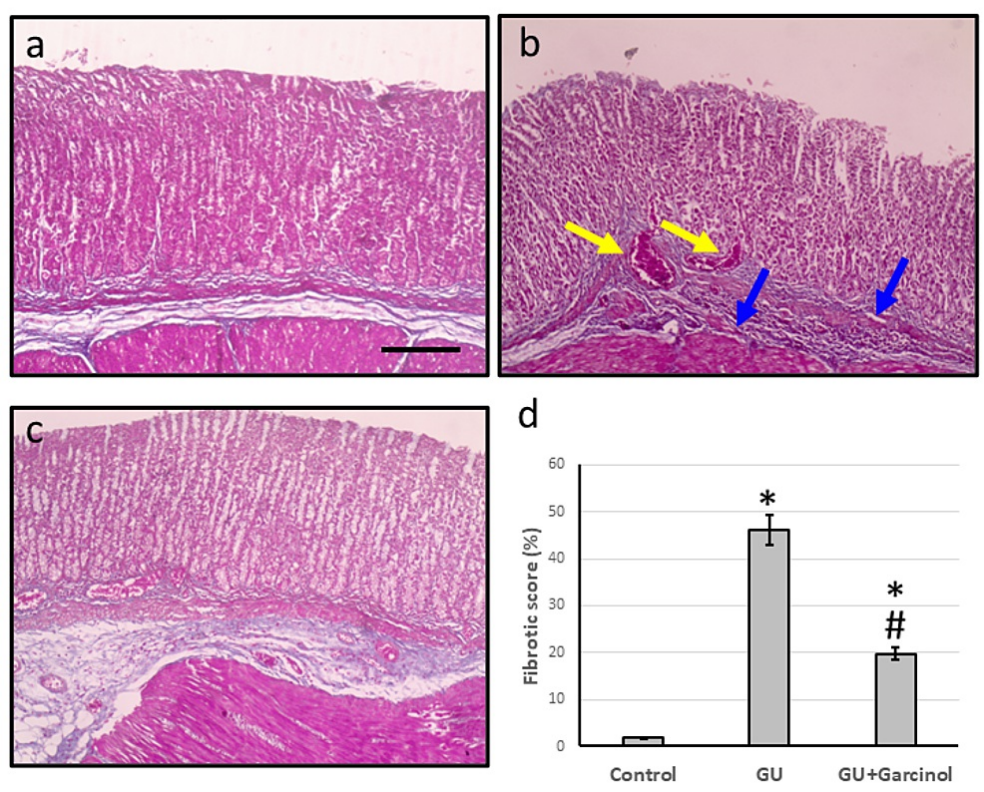
Gastric sections stained with Masson trichrome in the control group showing normal gastric tissues (a), the GU showing severe hemorrhage and extensive fibrosis (b), and the GU treated with 50 mg/kg garcinol group showing improvement in the structure of gastric tissues (c). The fibrotic score was determined by calculating it in 10 high field power fields and then presenting it as an average along with its standard deviation (d). Yellow arrows represented severe hemorrhage and blue arrows represented extensive fibrosis. ^*^Represented a significant difference for the group as compared with the control group at p<0.05. ^#^Represented a significant difference for the group as compared with the GU group at p<0.05. Scale bar: 100 μm. GU, gastric ulcer

Upon examining micro-images of the GU group stained with Masson trichrome, it was discovered that there was severe hemorrhage and extensive fibrosis present, resulting in an elevated fibrotic score when compared to the control group. However, the treatment of GU rats with garcinol was found to significantly reduce gastric tissue hemorrhage and fibrosis while also lowering the fibrotic score in comparison to the GU group, as shown in Figure 2.

Effect of garcinol on GU-induced elevation in DNA polymerization

**Figure 3:**
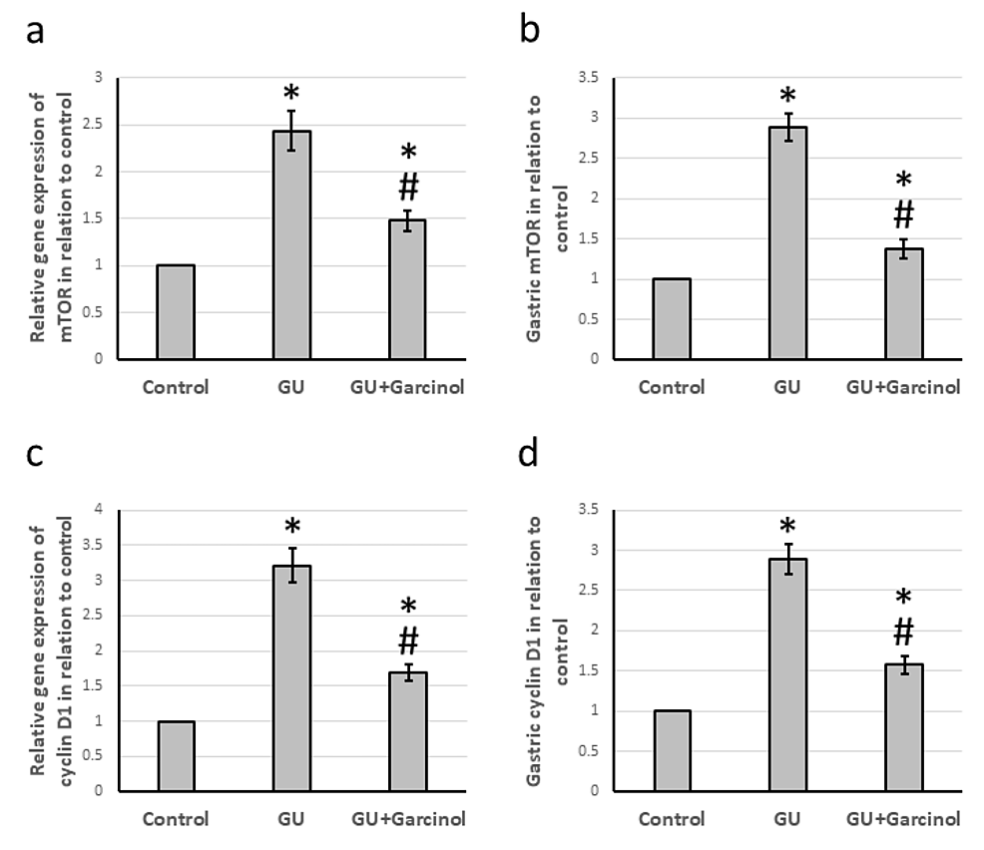
The effect of GU and administration of 50 mg/kg garcinol on the gene expression of mTOR (a) and cyclin D1 (c) associated with the gastric level of mTOR (b) and cyclin D1 (d). ^*^Represented a significant difference for the group as compared with the control group at p<0.05. ^#^Represented a significant difference for the group as compared with the GU group at p<0.05. GU, gastric ulcer; mTOR, mammalian target of rapamycin

Rats with GU exhibited elevated levels of gene expression and protein for mTOR and cyclin D1 compared to the control group. However, when treated with garcinol, the expression of TOR and cyclin D1 in GU rats significantly decreased but remained higher than in the control group, as shown in Figure 3.

Effect of garcinol on GU-induced elevation in cell proliferation 

**Figure 4:**
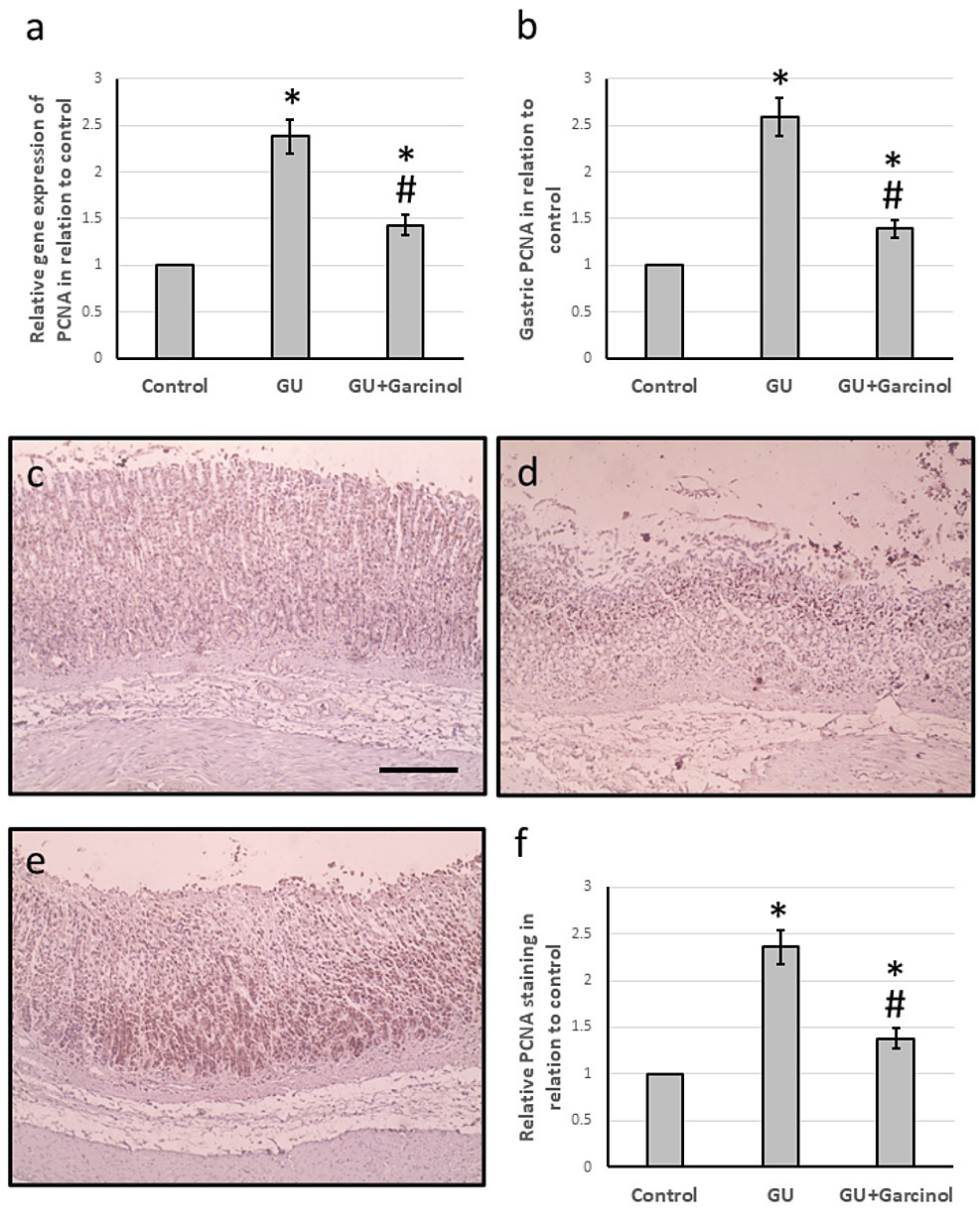
The effect of GU and administration of 50 mg/kg garcinol on the gene expression of PCNA (a) and the gastric protein levels of PCNA (b). Gastric sections were stained with anti-PCNA antibodies in the control group showing low staining levels (c), the GU group showing many areas highly stained (d) and the GU group treated with garcinol group with low staining areas (e). Immunohistochemistry score was calculated for positive staining in all groups and analyzed (f). To determine the immunohistochemistry score, the portion of the area that was stained with anti-PCNA was divided by the total tissue area in the field. This calculation was performed in 10 different fields for each section of the animal. ^*^Represented a significant difference for the group as compared with the control group at p<0.05. ^#^Represented a significant difference for the group as compared with the GU group at p<0.05. Scale bar is 50 µm. GU, gastric ulcer; PCNA, proliferating cell nuclear antigen

After conducting our investigation, we found that the group known as GU showed higher levels of both PCNA gene expression and protein levels when compared to the control group. Upon further analysis of micro-images that were stained with anti-PCNA antibodies, we discovered that there was a noticeable increase in staining areas in the GU rats as opposed to the control rats. However, we observed a significant reversal of these effects when the GU rats were treated with garcinol, as shown in Figure 4.

Effect of garcinol on GU-induced activation of inflammation pathway

**Figure 5:**
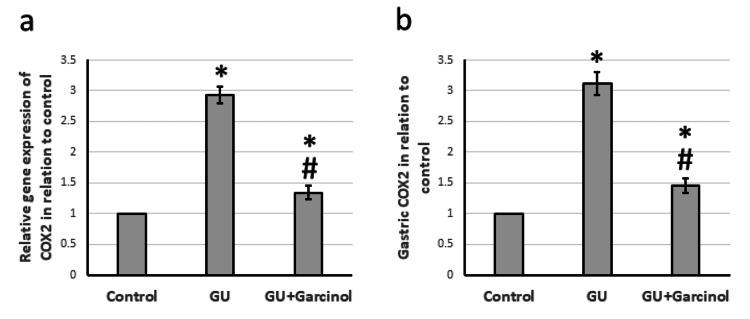
The effect of GU and administration of 50 mg/kg garcinol on the gene expression of COX2 (a) and its gastric level (b). ^*^Represented a significant difference for the group as compared with the control group at p<0.05. ^#^Represented a significant difference for the group as compared with the GU group at p<0.05. GU, gastric ulcer; COX2, cyclooxygenase-2

**Figure 6:**
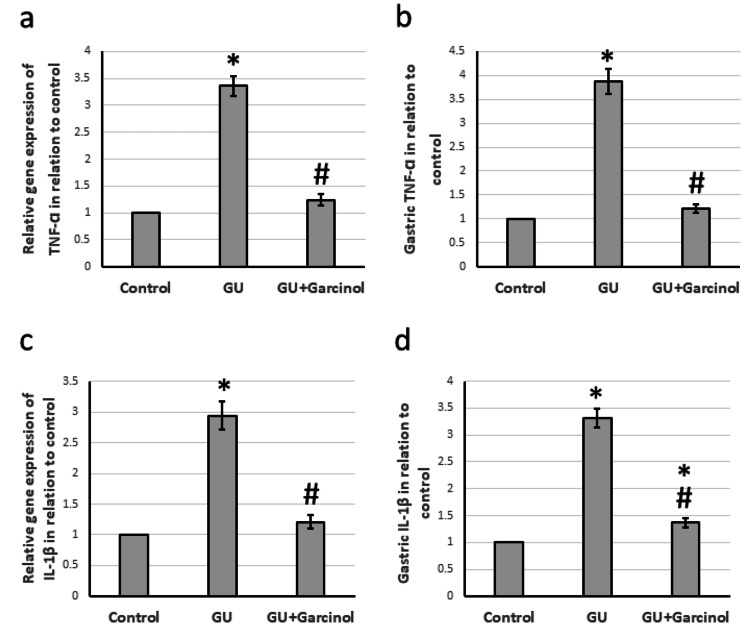
The effect of GU and administration of 50 mg/kg garcinol on the gene expression of TNF-α (a) and IL-1β (c) associated with the gastric levels of TNF-α (b) and IL-1β (d). ^*^Represented a significant difference for the group as compared with the control group at p<0.05. ^#^Represented a significant difference for the group as compared with the GU group at p<0.05. GU, gastric ulcer; IL-1β, interleukin-1β; TNF-α, tumor necrosis factor-α

**Figure 7:**
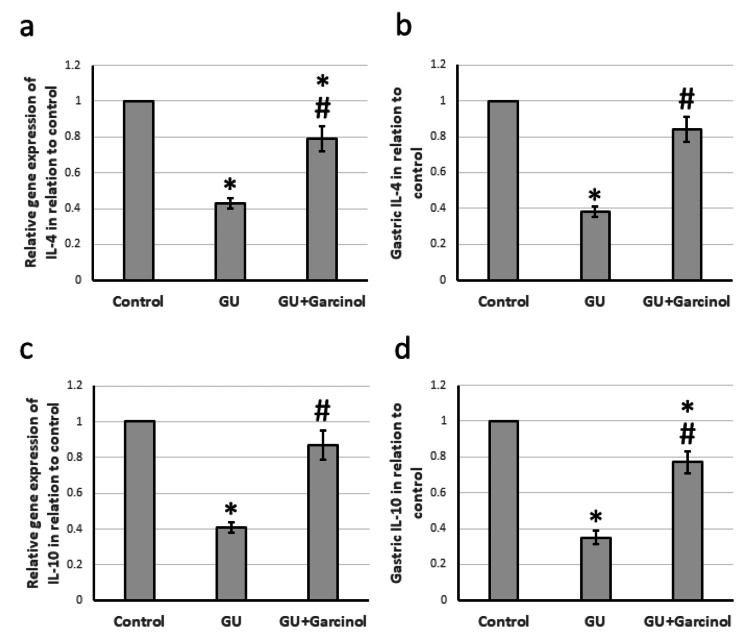
The effect of GU and administration of 50 mg/kg garcinol on the gene expression of IL-4 (a) and IL-10 (c) associated with the gastric level of IL-4 (b) and IL-10 (d). ^*^Represented a significant difference for the group as compared with the control group at p<0.05. ^#^Represented a significant difference for the group as compared with the GU group at p<0.05. GU, gastric ulcer; IL-4, interleukin-4; IL-10, interleukin-10

The research conducted on GU rats revealed that the COX2 gene and protein levels were higher in comparison to the control group, as shown in Figure 5. Along with this, GU resulted in increased gene expression and protein levels of TNF-α and IL-1β as depicted in Figure 6. Moreover, Figure 7 shows that there was a significant decrease in gene expression and gastric protein levels of both IL-4 and IL-10 in GU rats compared to control rats. However, when treated with garcinol, GU rats showed a decrease in the expression of COX2, TNF-α, and IL-1β, along with a significant increase in the expression of IL-4 and IL-10 compared to untreated GU rats. It is important to note that these findings were obtained by analyzing gastric samples from the rats and the results of the study indicate a positive effect of garcinol on the expression of inflammatory genes and proteins.

## Discussion

Gastric ulcer is a serious health condition that affects people all around the world. If left untreated, it can lead to severe complications that significantly impact the quality of life. Although many medications are available to treat GU, they often have unwanted side effects. In our study, we induced GU in rats using indomethacin and observed significant morphological changes, including hemorrhaging, ulceration, decreased gastric solution pH, and increased mucous production. Upon examination of micro-sections stained with Masson trichrome, we also observed severe hemorrhaging, extensive fibrosis, and elevated fibrotic scores. We chose garcinol, a compound known for its antioxidant and anti-inflammatory properties, for its potential therapeutic effects. Our findings showed that garcinol effectively improved the morphology of the rats' stomachs, elevated gastric solution pH, and reduced mucous production compared to untreated rats with GU. Garcinol was previously found to have attenuated indomethacin-induced GU in rats by reducing free radicals in gastric tissues [9]. We conducted this study to investigate the protective effects of garcinol on DNA polymerization markers (mTOR and cyclin D1), cell proliferation markers (PCNA), and inflammatory markers (COX2, TNF-α, and IL-1β).

Cell signaling is a complex process, and cyclins play a crucial role in it by interacting with cyclin-dependent kinases and participating in every phase of the cell cycle. Among these, cyclin D1 gene has been identified as a significant contributor to the transition of cells from G1 to S phase [18]. Similarly, the protein mTOR plays a crucial role in regulating cellular metabolism and is also important for the development of T cells. Additionally, it is a key regulator of cell growth and proliferation. It functions as a switch for anabolic and catabolic processes in cells [19]. Our research has shown that the levels of both mTOR and cyclin D1 were significantly elevated in the gastric tissues of GU rats, but treatment with garcinol helped ameliorate this. Garcinol has been previously found to reduce the expression of cyclin D1 in endometrial, gastric, and colon cancers [20-21], as well as inhibit the expression of mTOR in human prostate cancer cells and xenograft [22]. This is the first study to demonstrate garcinol's ability to inhibit the expression of both cyclin D1 and mTOR in GU.

The essential protein PCNA plays a crucial role in DNA replication, repair, chromatin organization, and transcription. It operates as a sliding clamp, polymerase switch factor, and recruitment factor, with its main function being the influence it has on various proteins involved in DNA synthesis, repair, and recombination [23]. Studies have shown that the content of PCNA in gastric epithelial cells is significantly higher in *Helicobacter pylori* infected biopsies from human patients, but it can be significantly reduced with treatment aimed at eradicating Helicobacter pylori [24]. In our research, we observed a significant increase in gene expression and gastric levels of PCNA, along with an increased area stained with anti-PCNA, in GU rats. However, treatment with garcinol significantly reduced the expression of PCNA, as it has been previously reported to do in azoxymethane-induced colonic aberrant crypt foci in male rats [25]. This is the first study to illustrate the ability of garcinol to inhibit the expression of PCNA in GU.

In cases of GU, inflammation plays a significant role. Non-steroidal anti-inflammatory drugs inhibit the production of prostaglandins from the arachidonic acid pathway, which is facilitated by COX isoenzymes. COX1 is a "housekeeping" enzyme constitutively expressed in the gastrointestinal tract, while COX2 predominates at sites of inflammation [26]. Studies have shown that deficiency of COX2 leads to delayed healing of GU, resulting in retardation of angiogenesis in the granulation tissue. Growth factors like EGF, bFGF, HGF, gastrin, and epiregulin can promote ulcer healing by up-regulating COX-2 expression [27]. On the other hand, proinflammatory cytokine TNF-α plays a role in the formation of GU by activating an acute inflammatory pathway leading to neutrophil infiltration in gastric mucosa. It downregulates gastric microcirculation and angiogenesis, which inhibits cell proliferation in ulcer areas, hindering ulcer healing. Therefore, deactivating TNF-α in the ulcer area enhances ulcer healing. Another inflammatory marker, IL-1β, is produced mainly by inflammatory cells such as monocytes/macrophages and plays a role in many inflammatory processes. It is increased in the gastric mucosa during GU and can cause recurrence of experimental GU in rats due to neutrophilic infiltration into scarred mucosa [28]. However, studies have shown that treatment of GU rats with garcinol attenuates the elevated expression of COX2, TNF-α, and IL-1β. Garcinol has been reported to reduce inflammation in many models, such as lumbar fifth spinal nerve ligation in rats, colon carcinogenesis in mice, and inflammatory bowel disease [20, 29]. However, no previous study has demonstrated whether garcinol can effectively inhibit inflammation in the GU.

Gastric ulcer occurs when the protective and destructive factors in the body are imbalanced. A few protective factors, including anti-inflammatory cytokines like IL-4 and IL-10, can counteract pro-inflammatory cytokines such as TNF-α, IL-1β, and IL-6, thus lessening damage to the gastric tissue. IL-4 is produced in response to Th-2 and regulates the immune system. It works by activating a specific receptor made up of α-chain and γc-chain, leading to the activation of the JAK-STAT pathway [30]. However, in GU rats, we observed a significant decrease in the expression of both IL-4 and IL-10. Treatment with garcinol, however, caused a significant increase in the expression of IL-4 and IL-10, which led to improved gastric tissue. This study is the first to demonstrate the ability of garcinol to elevate the expression of IL-4 and IL-10 in GU.

**Figure 8:**
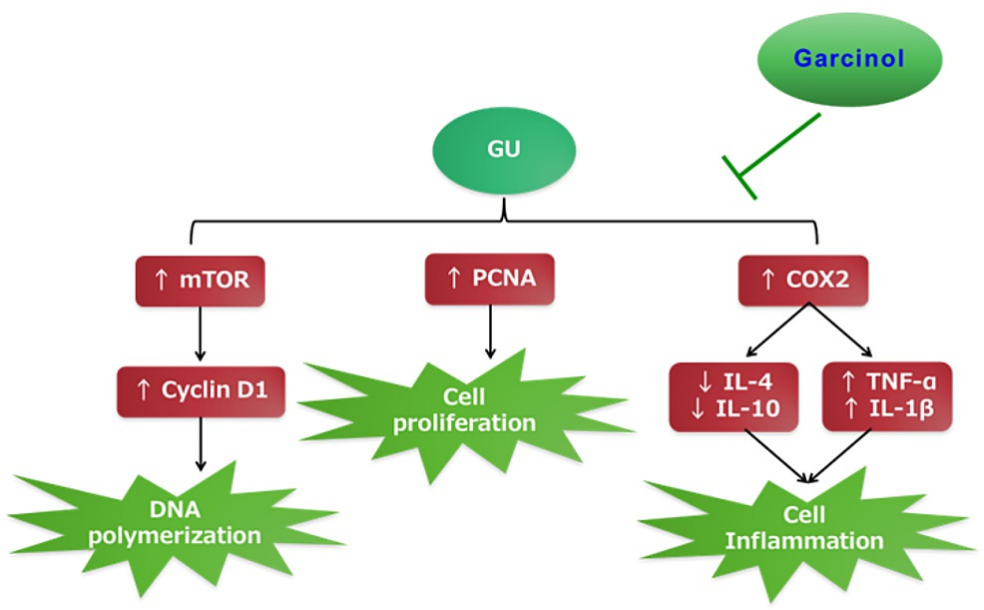
Mechanism of protective effects of garcinol in GU. COX2, cyclooxygenase-2; GU, gastric ulcer; IL, interleukin; mTOR, mammalian target of rapamycin; PCNA, proliferating cell nuclear antigen; TNF-α, tumor necrosis factor-α Image credits: The authors of the manuscript

It is worth noting that garcinol is a natural product. The mechanism of its therapeutic impact in Ehrlich solid carcinoma (ESC) is outlined in Figure 8. In our study, we used a dose of 50 mg/kg over a period of three weeks, which is lower than the 100 mg/kg/day dose that was given orally to rats for 28 days without any toxic effects [31]. Additionally, in our research, we administered garcinol orally, which is a form of protection that can be obtained by consuming garcinia fruits. The garcinia fruit contains 1.5%-3.67% garcinol [32], so, the dosage used in this study can be obtained by consuming garcinia without difficulty. Nevertheless, it is important to note some limitations of the current research. Rats and humans have different metabolic processes, which may result in different drug effects.

## Conclusions

In this experimental study on rats, garcinol was found to have therapeutic effects against GU. It was observed that garcinol restored normal gastric pH and reduced mucous production, reducing mucosal hemorrhagic lesions and fibrosis. The protective effects of garcinol can be attributed to its ability to decrease the expression of DNA polymerization markers, cell proliferation markers, and inflammatory markers at the gene and protein levels.
